# Evaluation of Starch as an Environmental-Friendly Bioresource for the Development of Wood Bioadhesives

**DOI:** 10.3390/molecules26154526

**Published:** 2021-07-27

**Authors:** Ana Arias, Gumersindo Feijoo, María Teresa Moreira

**Affiliations:** CRETUS, Department of Chemical Engineering, School of Engineering, Universidade de Santiago de Compostela, 15705 Santiago de Compostela, Spain; gumersindo.feijoo@usc.es (G.F.); maite.moreira@usc.es (M.T.M.)

**Keywords:** starch bioadhesives, chemical modification of starch, wood bioadhesives, life cycle assessment, environmental sustainability, formaldehyde-free wood adhesives

## Abstract

The environment is a very complex and fragile system in which multiple factors of different nature play an important role. Pollution, together with resource consumption, is one of the main causes of the environmental problems currently affecting the planet. In the search for alternative production processes, the use of renewable resources seeks a way to satisfy the demands of resource consumption based on the premises of lower environment impact and less damage to human health. In the wood sector, the panel manufacturing process is based on the use of formaldehyde-based resins. However, their poor moisture resistance leads to hydrolysis of amino-methylene bonds, which induces formaldehyde emissions throughout the lifetime of the wood panel. This manuscript investigates the environmental profile associated with different wood bioadhesives based on starch functionalization as a renewable alternative to formaldehyde resins. Considering that this is a process under development, the conceptual design of the full-scale process will be addressed by process modeling and the environmental profile will be assessed using life cycle assessment methodology. A comparative study with synthetic resins will provide useful information for modify their development to become real alternatives in the wood-based panel industry. The results obtained show the enormous potential of starch bioadhesives, as their environmental impact values are lower compared to those based on petrochemicals. However, certain improvements in the energy process requirements and in the chemical agents used could be developed to provide even better results.

## 1. Introduction

Formaldehyde is an aldehyde with high flammability and volatility potential, produced from the dehydrogenation and catalytic oxidation of methanol [1]. It is one of the most widely used crosslinking agents in the wood-based panel manufacturing industry, given its ease of processing, wide availability, low cost and high reactivity [2,3], representing a reference alternative in the wood production sector based on its technical and economic efficiency. However, environmental and health implications have to be taken into account as it is categorized in the REACH list as a carcinogen (category 1B) and mutagen (category 2). In particular, the main sources of hazard identified for formaldehyde are related to its atmospheric emissions and its potential harm to human health, in addition to the impacts caused by its production process (GHG emissions, consumption of nonrenewable fossil resources, toxicity, etc.). Given the reasons listed above, in recent years strict legal requirements have been developed in relation to the emission limits considered for formaldehyde, so that emission levels below 0.3 mg/L must be ensured [4] or by considering adhesives formulated with hardeners or scavengers that prevent or reduce the release of formaldehyde from the wood panel product [5,6,7,8,9].

The alternative based on renewable raw materials for the development of bioadhesives has been considered as an option of special interest. These include the use of soy [10,11,12], tannins [13,14,15], lignin [16,17,18], wood fibers [19,20], plant polymers [21,22] and starch [23,24,25]. In particular, starch is the second most abundant lignocellulosic polymer in nature [26]. Moreover, it is a low-cost resource with high potentiality, since it is biodegradable. However, despite these advantages, its direct use, i.e., as “native starch”, is not feasible for the development of bioadhesives for wood, since the large amount of hydrophilic hydroxyl groups in its molecular structure leads to low tolerance to moisture and high water absorption capacity [19]. In addition, it is necessary to provide active centers in its structure to improve adhesion strength and, in turn, control viscosity and morphological properties [27]. Therefore, a pretreatment is required to reduce the amount of hydroxyl groups present in their structure by adding crosslinking agents [26]. Thus, four bioadhesive alternatives will be evaluated in which different processing techniques have been considered, thus increasing their potential to be employed in the manufacture of wood-based panels. Considering the above, a large-scale design was carried out, including mass and energy balances based on laboratory data reported in the literature. A production capacity of 1000 kg/h of bioadhesive was considered, in which a production process analogous to that of the most commonly used synthetic resins (urea-formaldehyde, phenol-formaldehyde and melamine-urea-formaldehyde) can be established [28].

On the other hand, it is also important to study their potential from an environmental point of view. Once the input and output flows were estimated, the life cycle assessment (LCA) methodology was applied to evaluate the environmental impacts associated with each of the proposed starch bioadhesives [29]. For its application, it is necessary to define the inventory in which all the components included in the product/process are quantified, the system boundaries and the calculation methodology. Thus, a comparative analysis has been carried out with the most widely used synthetic-based resins, whose production processes are fully optimized. The evaluation of improvements or weaknesses in terms of the environmental impacts of their production processes has also been considered.

The objective of this research article is to evaluate four starch bioadhesives as alternatives to synthetic resins for the adhesion of wood-based panels, using a combination of process modeling and LCA methodology. The production capacity considered was 1000 kg/batch of bioadhesive and the functional unit was the production of 1 kg of bioadhesive, the basis of calculation to refer all inputs and outputs of the system.

The development of LCA studies involves a series of categorized and interrelated steps in a circular perspective: definition of goal and scope, inventory analysis, impact assessment and interpretation of results. The functional unit (FU) as defined by ISO is the quantified performance of a product system and its value must be consistent with the objective of the assessment and fully measurable [30]. The scope of the study has been selected within a “cradle-to-gate” approach. Thus, the LCA study is developed from the extraction of all necessary inputs in the process to the moment the product is ready for market. Therefore, transport activities, the use of the product by the consumer and the processes associated with its recycling, recovery or final disposal are outside the scope of the study. This approach has been considered appropriate as it allows not only for a comprehensive environmental analysis of the processes under development but also establishes a framework for completing environmental product declarations (EPDs) [31].

Regarding the database used for the analysis, Ecoinvent has been selected for conducting the life-cycle inventories, as it includes basic information on the main inputs, both material and energy, and outputs [32]. The calculation method considered for the development of the LCA was Recipe 2016, a methodology with a hierarchist perspective that includes both midpoint and endpoint indicators.

## 2. Materials and Methods

### 2.1. Description of the Four Bioadhesive Processes

Two main starch pretreatment methods have been considered for the analysis: hydrolysis and oxidation.

#### 2.1.1. Pretreatment Method 1: Starch Hydrolysis

The acid hydrolysis of starch leads to a reduction of its molecular weight, as it is broken down into its monomeric units: amylose and amylopectin. Specifically, amylose is hydrolyzed by cleavage of the α-1-4 bond and amylopectin by the α-1-4 bond [33], using HCl at low temperature, specifically, at a temperature below gelatinization [34]. In this way, a double objective is achieved, on the one hand, the formation of a greater number of active centers for the subsequent polymerization reactions with the crosslinking agents and, on the other hand, a reduction in viscosity, which is a decisive factor in the mechanical properties required for the application of the bioadhesive [35]. After acid hydrolysis, polymerization proceeds, which requires the development of a grafting process, based on the addition of monomeric units that will be joined by covalent bonds to the amylose and amylopectin units [36]. There are different grafting methods, the most widely used are free-radical grafting (FRG), as it is the simplest and cheapest way to modify biopolymers [37], in which grafting induced by chemical initiators has been chosen for its simplicity and efficiency. The most used initiating agents are sulfate salts (ammonium sulfate, ferrous sulfate), nitrate salts (ceric ammonium nitrate) and also Fenton chemicals [36]. After this first stage, the formation of reactive centers in the amylose and amylopectin polymers is achieved, to which the monomers selected for the adhesive formulation such as polyvinyl alcohol, vinyl acetate or butyl acrylate will be attached. In this way, a polymerization process takes place, thus improving the mechanical properties of the starch-based bioadhesive.

##### Alternative 1. Hydrolyzed Starch Bioadhesive with Bio-Oil

This alternative considers cassava starch, which has certain advantages over other starch sources, since it has a lower gelatinization temperature, which favors less energy-demanding processes, and also stands out for its structural stability and thermogravimetric properties [38,39]. After acid hydrolysis at a temperature of 60 °C, the grafting process proceeds, using (NH_4_)_2_SO_4_ as the initiating agent. The monomers that bind to the starch structure in the polymerization process are polyvinyl alcohol (PVA), which binds through hydrogen bonds [39], vinyl acetate and butyl acrylate, which bind through the free hydroxyl radicals of amylose and amylopectin [40]. After this first polymerization, a second one is carried out in the pursuit to improve the thermogravimetric properties of the starch bioadhesive. For this, it is required, again, to add an initiating agent, the same as for the first polymerization step (ammonium sulfate). Then, bio-oil is slowly added, which will react with the free hydroxyl groups of the amylose and amylopectin units and bind by the formation of ether bonds [41]. The reason for adding this bio-oil is that its presence in the structure of the bioadhesive improves its properties: greater stability and resistance to external agents [42], such as humidity or temperature variations.

##### Alternative 2. Starch Bioadhesive Hydrolyzed with N-Methylol Acrylamide

The second starch bioadhesive alternative is carried out based on three main steps in the formulation procedure: starch pretreatment based on acidolysis with HCl, followed by linear polymerization, considering the use of three polymers: sodium dodecyl sulfate (SDS), ammonium persulfate (APS) and vinyl acetate (VAc) for 3.5 h at 70 °C, and finally the formation of a network polymer, by the addition of N-methylol acrylamide (NMA), requiring 4.5 h and 85 °C [43]. Another aspect to take into account is the thermal variability that requires each stage of the process. While acidolysis starts at 60 °C, it subsequently evolves until it reaches 90 °C. In the case of the linear starch polymerization stage, the process starts at 70 °C and increases to 85 °C in the last 30 min to favor the process yield, and finally a decrease to 50 °C to obtain the final bioadhesive ready for application on wood panels. As for the type of bond formed between the starch polymer and N-methylol acrylamide, these are strong hydrogen bonds, which favors the improvement of the mechanical properties of the final bioadhesive, especially in terms of shear and water resistance [44].

#### 2.1.2. Pretreatment Method 2: Oxidation of Starch

Pretreatment by oxidation is also commonly employed for structural modification of the native starch molecule. However, it occupies the second position in terms of preference, since it can lead to partial depolymerization [45], which does not occur for acid hydrolysis. The result of this pretreatment is the formation of carboxylic groups or aldehydes from the oxidation of the primary and secondary hydroxyl groups of the glucose units that make up the amylose and amylopectin polymers [46]. The oxidation of native starch involves the loss of its crystallization, leading to the weakening of hydrogen bonds, which will facilitate the bonding of monomeric units in the subsequent polymerization process [47].

This oxidative process is going to require the presence of oxidizing chemicals, such as organic (i.e., NaClO) or inorganic (H_2_O_2_) peroxides, nitrogen (HNO_2_) or metal (CrO_3_) compounds, together with a catalyst, which will be transition metals in cationic form [46]. However, the choice of one or the other should focus not only on the performance of the oxidation process, but also on the environmental impacts they may generate. While hydrogen peroxide could be considered as the most “environmental-friendly”, the use of metal compounds as oxidizing agents would be the least suitable, from an environmental point of view, since the release of heavy metals into the environment leads to significant environmental impacts [48]. After this first oxidative pretreatment, the polymerization process proceeds, analogous to that presented above for the case of acid hydrolysis pretreatment.

##### Alternative 3. Starch Bioadhesive Oxidized with FeSO_4_ and H_2_O_2_

This third alternative is based on the development of a Fenton-type reaction, in which Fe^2+^ is oxidized to Fe^3+^ in the presence of hydrogen peroxide, which is transformed into a hydroxyl radical (Reaction 1). The presence of this radical will result in the oxidation of starch as it reacts with the hydroxyl groups of the glucose units, leading to the creation of carboxylic groups and aldehydes [49].

(1) $$H_{2}O_{2}+{\;\mathrm{Fe}}^{2+}\to {\;\mathrm{Fe}}^{3+}+{\;\mathrm{OH}}^{-}+{\;\mathrm{OH}}^{•}{}^{\;}$$

After this first pretreatment step, polymerization is carried out with the following chemicals: PVA, sodium dodecyl sulfate (SDS), ammonium persulfate (APS), silane coupling agent, butyl acrylate (BAc) and vinyl acetate (VAc) [50] due to a number of reasons: good adhesive strength [29], better dispersion [51,52], higher adhesion strength, better viscosity [53,54], water resistance [55] and thermal stability [56].

##### Alternative 4. Starch Bioadhesive Oxidized with NaClO and ECH

Unlike Alternative 3, in this last starch bioadhesive option, the oxidative pretreatment considers the use of NaClO. In this case, the hypochlorite ion (ClO^−^) oxidizes the starch molecule by removing the hydrogen atom from the hydroxyl groups and the consequent formation of the carboxylic or ketone groups (Reaction 2). With this transformation of the molecular structure of starch, an increase in the polarity of the molecule is achieved and a greater facility for the development of the grafting process and subsequent polymerization [57].

(2) $$\mathrm{Starch}-\mathrm{OH}+{\;\mathrm{OCl}}^{-}\to \;\mathrm{Starch}=O+{\;H}_{2}O+{\;\mathrm{Cl}}^{-}{}^{\;}$$

In this study, in addition to including NaClO in this first stage of activation, it also utilizes epichlorohydrin (ECH) [58], which binds to the starch molecule through the formation of diether bridges developing a crosslinking reaction [59]. PVA, sodium lauryl sulfate (LSS), Tween 80, APS and VAc are also included in the formulation of this bioadhesive. The advantages of using LSS as an emulsifying compound are based on an improvement in the stability of the adhesive compound, in addition to an increase in shear strength [60]. As for Tween 80, it is a surfactant used to reduce surface tension and improve wetting in the board–adhesive bonding process [61].

### 2.2. System Boundaries

**Figure 1:**
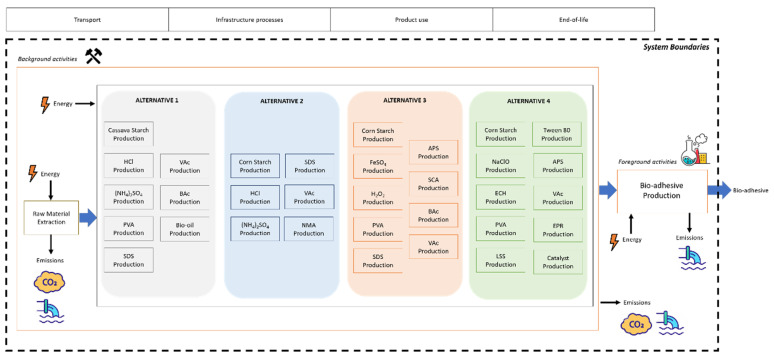
System boundaries of the bioadhesives’ production considered for LCA analysis.

All the stages regarding the extraction of raw materials, the production of the bioadhesive and the emissions and waste management have been considered within the system boundaries of the LCA analysis (Figure 1). On the other hand, transport activities and infrastructure process were excluded and, as it has been considered a cradle-to-gate approach for the assessment, also the bioadhesive’s uses and its end-of-life stages are out of the system boundaries.

### 2.3. LCA Inventories

The inventories considered for each of the starch bioadhesive alternatives are shown in Table 1, Table 2, Table 3 and Table 4, including the inputs required from the technosphere, both materials and energy requirements, and the outputs to the technosphere, which comprise the main product, the bioadhesive, and the emissions associated with its production.

### 2.4. LCA Parameters

The calculation methodologies selected to perform the life cycle assessment of the starch bioadhesives were the following: ReCiPe Midpoint (H) V1.03 World (2010), ReCiPe Endpoint (H/H) V1.03 World (2010) and USEtox V1.04 Europe (2004). The impact categories considered for study in this article are displayed on Table 5.

## 3. Results and Discussion

### 3.1. Environmental Profiles of Starch Bioadhesive Alternatives

#### 3.1.1. Alternative 1. Hydrolyzed Starch Bioadhesive with Bio-Oil

**Figure 2:**
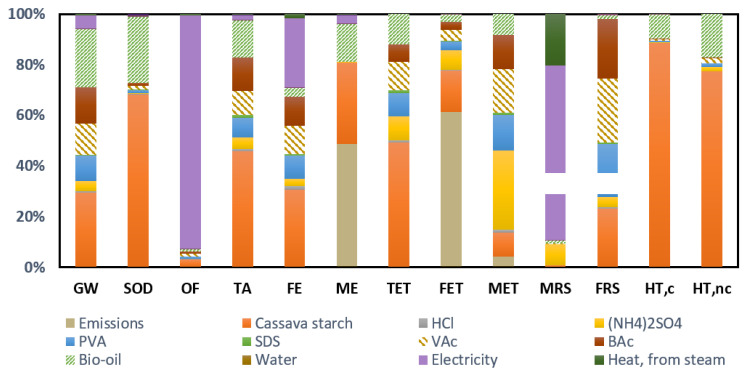
Environmental profile of the hydrolyzed starch bioadhesive with bio-oil (Alternative 1).

The environmental impacts are shown in Table 6 and the profile of this starch bioad-hesive alternative is shown in Figure 2.

There is some variability in the environmental contribution according to the impact categories, although three main hotspots can be identified: the production of the cassava starch, the electricity requirements and the emissions from the bioadhesive formulation. The reason for the significant environmental contribution of starch is attributed to cassava cultivation due to the direct emissions of CO_2_, ammonia and nitrates as a consequence of the use of fertilizers in the crop field and the generation of crop residues, and the use of diesel fuel for agricultural machinery.

As for emissions, they are characterized by ammonium compounds, specifically ammonium chloride and ammonium sulfate, as well as sulfuric acid. While ammonium species cause eutrophication [62], the implication of sulfuric acid can cause variations in the pH of the aquatic environment, with a moderate toxicity potential [63].

The impact of the electricity needs of the production process of this bioadhesive alternative is noteworthy in the MRS and OF categories. The reason for its contribution in the MRS category is associated with the production of electricity from nonrenewable fossil resources. As for the OF category, energy consumption involves the formation of atmospheric ozone as a product of the reaction between nitrogen oxides and volatile organic compounds, when exposed to sunlight [64].

#### 3.1.2. Alternative 2. Hydrolyzed Starch Bioadhesive with N-Methyl Acrylamide

**Figure 3:**
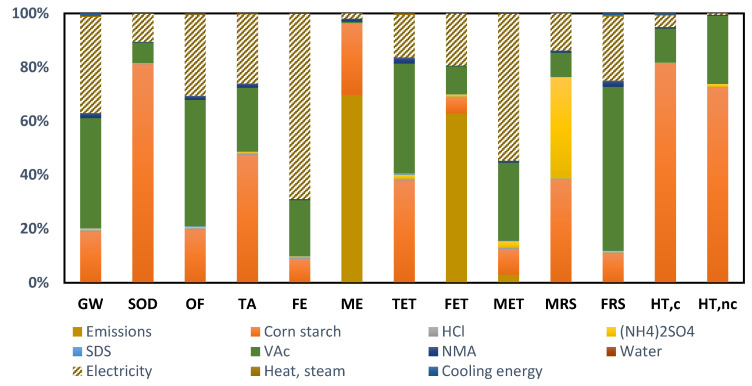
Environmental profile of the hydrolyzed starch bioadhesive with N-methyl acrylamide (Alternative 2).

A certain analogy can be observed in the environmental profile of this option compared to the results shown previously (Table 7 and Figure 3).

Although there is some contribution from starch, VAc and direct emissions, there is a greater environmental influence from electricity consumption. If the inventory of this process is analyzed, it can be noted that the electricity requirements per kg of bioadhesive produced is 10 times higher (the processing time is also longer, being in this case 12 h of batch process, compared to 7 h for the first alternative studied). This is the reason why the contribution of electricity in the environmental profile is more noticeable for this second bioadhesive option. A promising and sustainable way to improve this environmental profile would be to consider the use of renewable energies, which would not only avoid the depletion of fossil resources but would also reduce the emissions associated with the raw material extraction processes and the production process itself.

#### 3.1.3. Alternative 3. Starch Bioadhesive Oxidized with FeSO_4_ and H_2_O_2_

**Figure 4:**
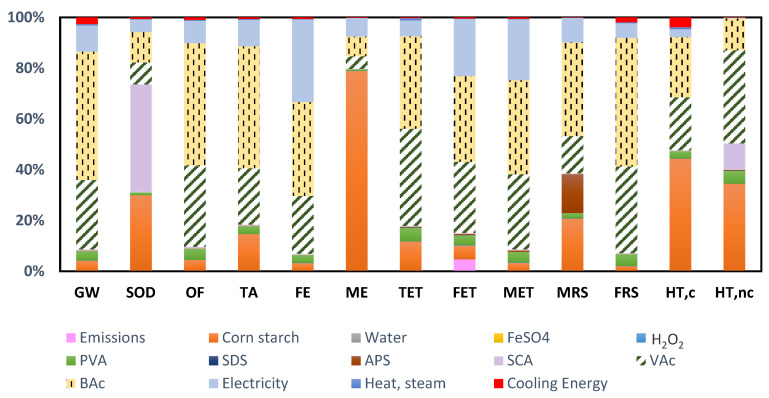
Environmental profile of the starch bioadhesive oxidized with FeSO_4_ and H_2_O_2_ (Alternative 3).

Both butyl acrylate (BAc) and vinyl acetate (VAc) account for more than 50% of the environmental contribution in most of the impact categories of this third alternative (Table 8 and Figure 4), with the exception of the SOD, ME and HT categories, where a higher impact influence of corn starch is perceived.

The background manufacturing activities of BAc and VAc are the reason for these high contributions on the environmental profile for this third bioadhesive alternative. As for BAc, it is produced by the esterification reaction of acrylic acid with methanol. Developing the LCA of its production, it is observed that methanol and the caloric requirements of the production process are the main hotspots of the environmental profile. On the other hand, when performing an in-depth analysis, regarding the methanol manufacturing process, which is based on the hydroformylation of propylene, the main contributors on its environmental profile are carbon monoxide, propylene and energy requirements. Therefore, this detailed analysis of BAc background activities allows us to identify that its contribution to the environmental profile of the starch bioadhesive is the result of the use of chemicals (methanol, carbon monoxide and propylene), which are the cause of the high contribution in categories such as GW, OF, TA, TET, FET and MET. On the other hand, thermal energy needs, obtained from nonrenewable fossil resources, are the cause of the impact in the MRS and FRS categories.

A similar procedure has been carried out to investigate the high contribution of VAc in the environmental profile obtained. The conclusion grasped after an exhaustive analysis of each of the stages of its production process, based on the reaction between ethylene and acetic acid, is that the chemicals with the greatest impact on the environment are acetic acid, carbon monoxide and methanol (mainly affecting the impact categories of ecotoxicity, eutrophication and climate change) and, on the other hand, the energy requirements, which contribute to the categories of scarcity of resources, both fossil and mineral.

#### 3.1.4. Alternative 4. Starch Bioadhesive Oxidized with NaClO and ECH

**Figure 5:**
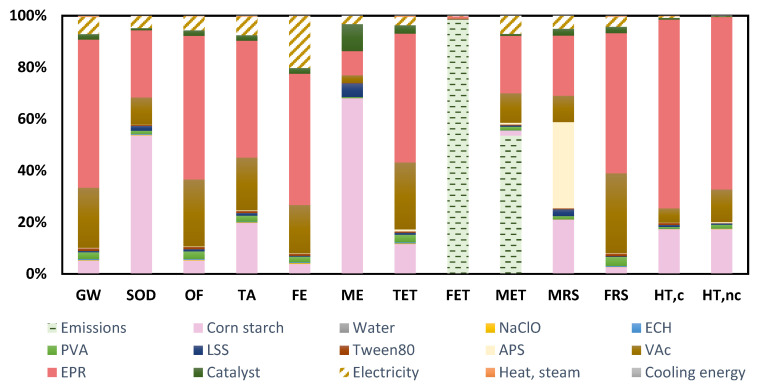
Environmental profile of the starch bioadhesive oxidized with NaClO and ECH (Alternative 4).

Specific details on the different impact categories are shown in Table 9. On the other hand, Figure 5 represents the environmental profile of the starch bioadhesive oxidized with NaClO and ECH.

As can be seen, two main items stand out in most of the impact categories: epoxy resin and corn starch, except for FET, MET and MRS. Regarding the FET and MET categories, the emissions released within the bioadhesive production process are the ones with the highest environmental contribution. The use of NaCl, HCl and H_2_SO_4_ is the reason for the high impact obtained in the categories of freshwater and marine ecosystem ecotoxicities. A suitable strategy of neutralization would allow to reduce the environmental contribution on these impact categories. As for corn starch, its influence on the environmental profile is the result of background activities, as analyzed in the previous profiles obtained for the other bioadhesive alternatives proposed here: fertilizer use in cultivation, energy use for machinery, among others.

Looking for the reason for such a high environmental contribution of the use of epoxy resin, the elements that make up its production process have been analyzed to determine if this high impact is the result of the use of chemical agents or if, on the contrary, it is due to the energy demand of its industrial production. Epoxy resin is produced from the reaction between bisphenol A and epichlorohydrin, both chemicals are obtained from non-renewable fossil resources and are the main hotspots identified in the environmental profile. Regarding bisphenol A, it is produced by catalytic condensation between phenol and acetone. To evaluate the reason for its high impact, a detailed analysis of its manufacturing process is carried out, based on the Hock process [65], an autocatalytic and exothermic oxidation process that uses cumene as raw material, identifying cumene as the main hot spot in the environmental profile obtained. Thus, a final analysis was carried out for this chemical agent, obtained from the alkylation of benzene and propene and, once again, it was observed that the elements of the inventory that lead to a greater environmental impact are the chemical agents used for its production, given its nonrenewable nature. As for epichlorohydrin, the conclusions obtained by performing an analysis similar to the previous one are the same, although, in addition to the identification of chemical agents as the main contributors to the environmental impacts generated, the emissions associated with the production processes also have a significant influence, given that its most widespread form of waste management is through incineration, which gives rise to emissions of hazardous agents, such as carbon dioxide, methane, nitrates and phenolic compounds, among others.

Therefore, after this exhaustive analysis, it is concluded that the chemicals necessary for the formulation of this starch bioadhesive alternative are the main causes of environmental impacts.

### 3.2. Comparison between Starch Bio-Based Adhesive Alternatives

**Figure 6:**
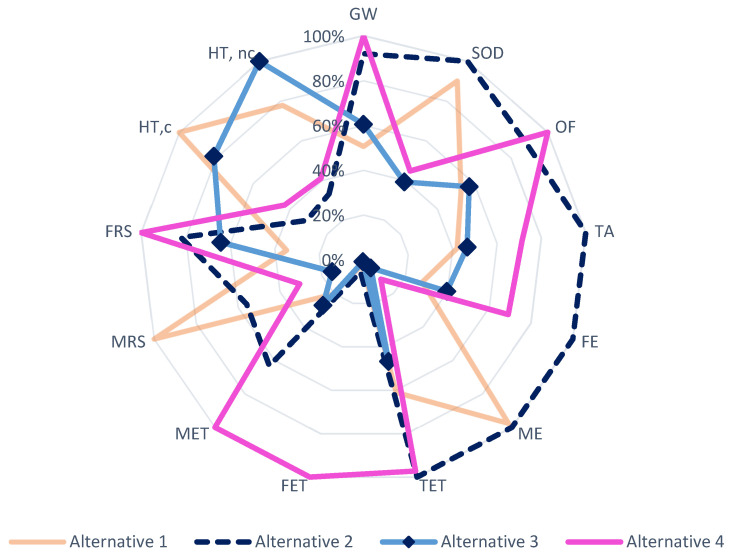
Comparative analysis of the starch bioadhesives alternatives.

The impact results obtained after the environmental assessment have been used for the comparison of the four starch-based bioadhesive alternatives proposed by applying the Recipe Midpoint and USEtox calculation methodologies. As can be seen in Figure 6, Alternative 2 (starch bioadhesive hydrolyzed with N-methyl acrylamide) is the one with the highest potential environmental impact, being the most detrimental in most of the impact categories related to environmental quality. On the other hand, in the two categories specific for damage to human health, it is identified as the best alternative, since it has the least negative impact on health.

The second worst alternative, from an environmental point of view, is Alternative 4 (starch bioadhesive oxidized with NaClO and ECH). In the ecotoxicity categories (FET, TET and MET), as well as in the GW and FRS categories, it has the highest impact values compared to the other three. The reason for its high contribution in these categories is based on the use of EPR as a crosslinking agent. In addition to requiring a significant amount per kg of bioadhesive produced (0.22 kg/kg), its production process is highly dependent on nonrenewable fossil resources, and also uses certain chemical agents with high potential negative impacts on environmental quality, resulting in significant environmental contributions.

The best results were obtained for Alternatives 1 and 3, Alternative 1 (hydrolyzed starch bioadhesive with bio-oil) being the most promising in most of the impact categories studied, with the exception of MRS, ME and HT, c. The reason for its significant contribution to the MRS category derives from the energy requirements of the adhesive formulation process since five different temperatures are required throughout its 12 h batch process, which will entail a significant consumption of nonrenewable fossil resources. As for its impact on the ME category, it is the result of the emission of chlorinated compounds, since these emissions are higher than those of the other bioadhesive alternatives proposed. Finally, in the HT, c impact category (Figure 2), the background activities associated with corn starch are the hotspot in this category. Since this second alternative has the highest ratio of starch/bioadhesive of the four proposed alternatives (0.44:1), its environmental impact in this USEtox category is also the most significant.

The results obtained for Alternative 3 also show the high potential of this bioadhesive from an environmental sustainability point of view, as its life cycle assessment has resulted in low impact values for most of the categories studied, except for HT, nc, where it is identified as the bioadhesive alternative with the highest impact. The use of VAc and BAc in its formulation is what leads to the emerging toxicity of this third alternative. Therefore, one way to reduce its impact, and thus improve its quality and consider it a safe option for human health, would be to use other polymerization agents, such as PVA, which has shown low levels of impact in different environmental categories.

### 3.3. Comparison with Synthetic Resins

To analyze whether bio-based starch bioadhesives are potential options to substitute synthetic resins, it is important to perform a comparative analysis between conventional processes and those under development. It must be considered that the fact of being called bio-based does not necessarily imply that the associated environmental impacts are always lower, taking into account that the large-scale production process has yet to be optimized. Therefore, obtaining lower impact results compared to synthetic resins would be an important stage in the field of bioadhesives as it implies their great opportunity of application and presence in the market, given their wide range of adaptability, improvement and refinement, since their manufacturing processes are still in a first degree of development.

**Figure 7:**
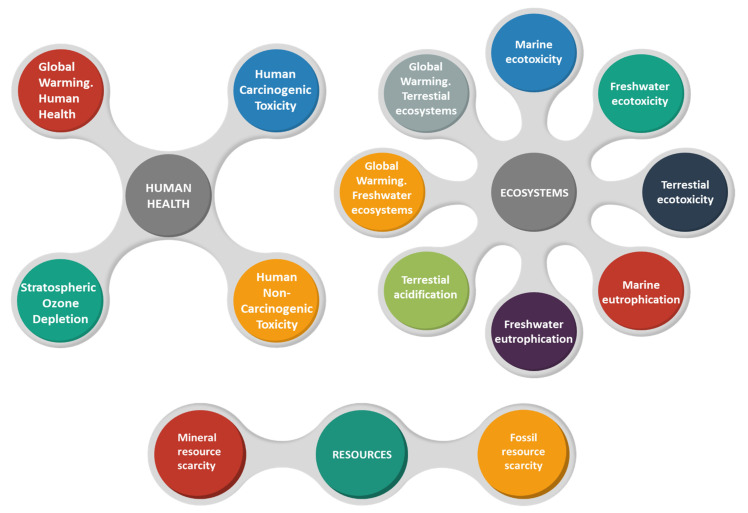
Endpoint characterization factors with midpoint categories and single score value calculation procedure.

To perform this comparison between starch bioadhesives and synthetic resins (UF, PF and MUF), the Endpoint V1.03 method has been used. The inventory data to develop the LCA of petrochemical adhesives have been taken from the Ecoinvent database (for UF and PF) and from Silva et al. (2015) [66]. The application of this calculation methodology provides three final scores, embodied under the categories of human health, ecosystems and resources. These three scores encompass midpoint categories by considering particular endpoint characterization factors, which are shown in Figure 7.

In this way, the environmental profiles of the proposed adhesive alternatives can be viewed in a more condensed and simple manner, thus facilitating their final comparison. In addition, by applying normalization factors, it is possible to obtain a final value, known as a single score, which includes the three categories mentioned, thus providing a global view of the impact caused by the development of each of the options proposed in this article.

**Figure 8:**
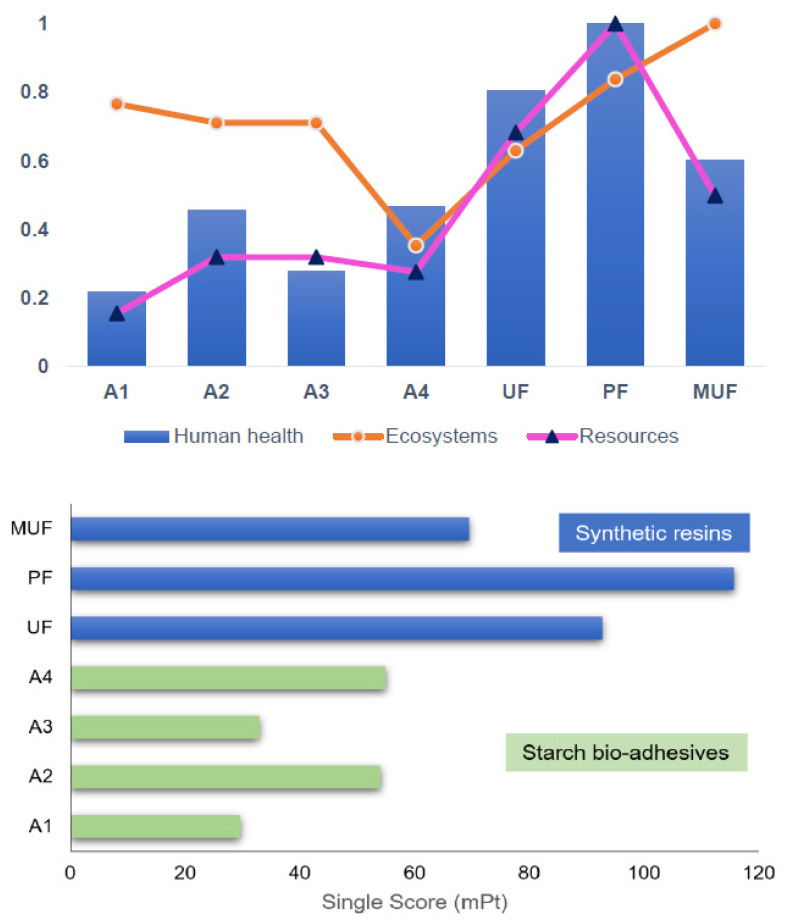
Endpoint characterization values obtained for the starch bioadhesives and synthetic resins.

The results obtained are plotted in Figure 8 so that the upper figure shows the endpoint scores for the starch bioadhesives and the synthetic resins, and the lower figure shows the single score values.

Outstanding results were obtained for the starch bioadhesives proposed in this article, comparing their final scores with those of the synthetic resins. All bioadhesives present lower impact values in the human health and resource scarcity categories, with Alternatives 1 and 3 standing out, Alternative 1 being the one that achieved a better environmental result, as it is the starch bio-based adhesive alternative with the lowest single score value. On the other hand, higher impact scores have been obtained in the ecosystems category, although with values analogous to those of the PF synthetic resin and lower than those obtained for the MUF, but not by a value that implies that the proposed starch bioadhesives can be neglected.

The individual scores of the synthetic resins are considerably higher than those of the starch bioadhesives. According to the values obtained, MUF is the most environmentally friendly petrochemical-based resin alternative, as it presented the lowest single score value and, therefore, its production process entails a lower degree of environmental impact. However, when compared to the results obtained for the bio-based resins, the single score of MUF is even higher than all of them. This could be considered as an indicator of the high applicability potential of starch bioadhesives to replace synthetic ones, given that their formulation processes provide a significantly lower environmental contribution, the reduction of the consumption of nonrenewable resources, the use of agroindustrial waste streams and a more favorable impact in terms of human health.

## 4. Conclusions

In this report, the life cycle assessment methodology has been selected to evaluate the environmental performance of four starch-based bioadhesive alternatives as possible substitutes for the most widespread synthetic resins for wood-based panels: UF, PF and MUF. In order to develop the necessary inventories for the application of the above methodology, a first large-scale simulation design was required, considering the experimental data available in the literature. The results obtained showed the enormous potential of starch bioadhesives, in terms of low environmental impacts caused by their production process compared to petrochemicals. Single score values of starch-based bioadhesives (Alternative 1: 30 mPt, Alternative 2: 54 mPt, Alternative 3: 33 mPt and Alternative 4: 55 mPt) are significantly lower than the ones obtained for formaldehyde-based adhesives, with 93, 116 and 70 mPt values for UF, PF and MUF adhesives, respectively.

However, according to the environmental profiles analyzed for each of the bio-based alternatives, certain improvements could be developed to provide even better results: optimization of energy requirements and reduction of certain chemical agents, due to their toxicity (i.e., BAc and VAc).

Thus, further research could be developed to further improve this proposed wood adhesive alternative from an environmental point of view. In addition, it would be desirable to develop research based on considering the durability and longevity of bio-based starch adhesives, in order to provide knowledge on appropriate and compatible treatments to be applied to wood-based panels to ensure their quality and strength. The hotspots identified in the environmental profiles could be useful for researchers and policy makers to move forward towards the framework of sustainable and formaldehyde-free wood adhesive alternatives.

## Figures and Tables

**Table 1:** Inventory data for the production of hydrolyzed starch bioadhesive with bio-oil (Alternative 1). Detailed data per functional unit (1 kg of bioadhesive). Acronyms: polyvinyl alcohol (PVA), sodium dodecyl sulfate (SDS), vinyl acetate (VAc), butyl acrylate (BAc).

Inputs from Technosphere			Outputs to Technosphere		
Materials			Products		
Cassava starch	0.354	kg	Bioadhesive	1	kg
HCl	0.010	kg			
(NH_4_)_2_SO_4_	0.022	kg			
PVA	0.042	kg	Emissions to water		
SDS	0.008	kg	NH_4_Cl	0.014	kg
VAc	0.051	kg			
BAc	0.034	kg	H_2_SO_4_	0.013	kg
Bio-oil	0.088	kg			
Water	0.439	kg	H_2_O	0.017	kg
Energy					
Electricity	0.12	kWh	(NH_4_)_2_SO_4_	0.005	kg
Heat	58.76	kJ			

**Table 2:** Inventory data for the production of hydrolyzed starch bioadhesive with N-methylol acrylamide (NMA) (Alternative 2). Detailed data per functional unit (1 kg of bioadhesive). Acronyms: sodium dodecyl sulfate (SDS), vinyl acetate (VAc), N-methyl acrylamide (NMA).

Inputs from Technosphere			Outputs to Technosphere		
Materials			Products		
Corn starch	0.442	kg	Bioadhesive	1	kg
HCl	16.146	g			
(NH_4_)_2_SO_4_	5.087	g			
SDS	5.773	g	Emissions to water		
VAc	0.310	kg	NH_4_Cl	6.732	g
NMA	11.059	g			
Water	0.496	kg	H_2_SO_4_	32.938	g
		
Energy			HCl	94.576	g
Electricity	1.453	kWh			
Heat, steam	86.63	kJ	NaCl	0.151	kg
Cooling energy	94.52	kJ			

**Table 3:** Inventory data for the production of starch bioadhesive oxidized with FeSO_4_ and H_2_O_2_ (Alternative 3). Detailed data per functional unit (1 kg of bioadhesive). Acronyms: polyvinyl alcohol (PVA), sodium dodecyl sulfate (SDS), ammonium persulfate (APS), silane coupling agent (SCA), vinyl acetate (VAc), butyl acrylate (BAc).

Inputs from Technosphere			Outputs to Technosphere		
Materials			Products		
Corn starch	0.064	kg	Bioadhesive	1	kg
Water	0.636	kg			
FeSO_4_	0.636	g			
H_2_O_2_	0.636	g	Emissions to water		
PVA	0.019	kg	FeH_3_	0.234	g
SDS	0.636	g			
APS	0.636	g	H_2_SO_4_	0.402	g
SCA	0.318	g			
BAc	0.136	kg			
VAc	0.143	kg			
Energy					
Electricity	0.272	kWh			
Heat, steam	62.92	kJ			
Cooling energy	197.98	kJ			

**Table 4:** Inventory data for the production of starch bioadhesive oxidized with NaClO and ECH (Alternative 4). Detailed data per functional unit (1 kg of bioadhesive). Acronyms: epichlorohydrin (ECH), polyvinyl alcohol (PVA), lauryl sodium sulfate (LSS), ammonium persulfate (APS), vinyl acetate (VAc), epoxy resin (EPR).

Inputs from Technosphere			Outputs to Technosphere		
Materials			Products		
Corn starch	0.131	kg	Bioadhesive	1	kg
Water	0.411	kg			
NaClO	1.969	g			
ECH	2.626	g	Emissions to water		
PVA	0.022	kg	NaCl	0.827	g
LSS	2.740	g			
Tween80	4.110	g	HCl	1.119	g
APS	2.814	g			
VAc	0.192	kg	H_2_SO_4_	0.912	g
EPR	0.221	kg			
Catalyst	0.012	kg			
Energy					
Electricity	0.288	kWh			
Heat, steam	58.39	kJ			
Cooling energy	18.16	kJ			

**Table 5:** Impact categories selected for performing LCA, including its acronyms and units.

Method	Impact Category	Acronym	Unit
ReCiPe Midpoint (H)	Global Warming	GW	kg CO_2_ eq
	Stratospheric Ozone Depletion	SOD	mg CFC_11_ eq
	Ozone Formation	OF	g NO_x_ eq
	Terrestial Acidification	TA	g SO_2_ eq
	Freshwater Eutrophication	FE	g P eq
	Marine Eutrophication	ME	g N eq
	Terrestial Ecotoxicity	TET	kg 1,4-DCB
	Freshwater Ecotoxicity	FET	g 1,4-DCB
	Marine Ecotoxicity	MET	g 1,4-DCB
	Mineral Resource Scarcity	MRS	g Cu eq
	Fossil Resource Scarcity	FRS	kg oil eq
ReCiPe Endpoint (H/H)	Human Health	HH	mPt
	Ecosystems	E	mPt
	Resources	R	mPt
USEtox	HT, c	HT, c	CTUh
	HT, nc	HT, nc	CTUh

**Table 6:** Environmental characterization of hydrolyzed starch bioadhesive with bio-oil.

Impact Category	Unit	Total	Emissions	Cassava Starch	HCl	(NH_4_)_2_SO_4_	PVA	SDS	VAc	BAc	Bio-oil	Water	Electricity	Heat, from Steam
GW	kg CO_2_ eq	0.92		0.27	5.08 × 10^−3^	0.03	0.09	4.65 × 10^−3^	0.11	0.13	0.21	0.1 × 10^−3^	0.05	6.06 × 10^−3^
SOD	mg CFC_11_ eq	2.92		1.99	6.45 × 10^−3^	0.01	0.03	1.83 × 10^−3^	0.04	0.04	0.77	5.74 × 10^−5^	0.03	1.48 × 10^−3^
OF	g NO_x_ eq	2.05		0.89	0.01	0.07	0.22	0.01	0.27	0.25	0.23	0.19 × 10^−3^	25.41	0.14
TA	g SO_2_ eq	3.67		1.64	0.03	0.15	0.28	0.05	0.34	0.46	0.52	0.42 × 10^−3^	0.09	6.98 × 10^−3^
FE	g P eq	0.24		0.08	3.48 × 10^−3^	7.67 × 10^−3^	0.02	1.92 × 10^−3^	0.03	0.03	9.04 × 10^−3^	9.05 × 10^−5^	0.07	4.52 × 10^−3^
ME	g N eq	2.17	1.10	0.73	0.38 × 10^−3^	0.49 × 10^−3^	1.66 × 10^−3^	0.27 × 10^−3^	2.01 × 10^−3^	2.00 × 10^−3^	0.33	6.71 × 10^−6^	0.09	7.16 × 10^−3^
TET	kg 1,4-DCB	1.37		0.66	0.01	0.13	0.12	0.02	0.15	0.09	0.16	8.67 × 10^−5^	0.19 × 10^−3^	1.36 × 10^−5^
FET	g 1,4-DCB	33.49	19.76	5.28	0.09	2.52	1.12	0.06	1.36	1.05	0.95	2.59 × 10^−3^	0.05	0.57 × 10^−3^
MET	g 1,4-DCB	12.75	0.47	1.02	0.14	3.43	1.54	0.09	1.87	1.49	0.91	3.61 × 10^−3^	3.44 × 10^−3^	3.76 × 10^−5^
MRS	g Cu eq	4.40		0.33	3.59 × 10^−3^	3.51	0.03	3.14 × 10^−3^	0.04	0.06	0.40	0.27 × 10^−3^	29.04	8.59
FRS	kg oil eq	0.28		0.06	1.65 × 10^−3^	0.01	0.05	1.63 × 10^−3^	0.07	0.06	3.85 × 10^−3^	2.69 × 10^−5^	1.27 × 10^−3^	1.84 × 10^−5^
HT, c	10^−10^ CTUh	7.14		6.32	4.39 × 10^−3^	0.01	0.04	2.36 × 10^−3^	0.05	0.03	0.67	2.36 × 10^−5^	7.82 × 10^−3^	3.87 × 10^−3^
HT, nc	10^−10^ CTUh	10.17		7.86	0.01	0.18	0.15	6.33 × 10^−3^	0.18	0.04	1.75	1.66 × 10^−5^	1.75 × 10^−3^	0.28 × 10^−3^

**Table 7:** Environmental characterization of hydrolyzed starch bioadhesive with N-methylol acrylamide.

Impact Category	Unit	Total	Emissions	Corn starch	HCl	(NH_4_)_2_SO_4_	SDS	VAc	NMA	Water	Electricity	Heat, Steam	Cooling Energy
GW	kg CO_2_ eq	1.67		0.32	8.21 × 10^−3^	9.14 × 10^−3^	3.35 × 10^−3^	0.68	0.03	1.13 × 10^−4^	0.60	8.93 × 10^−3^	0.01
SOD	mg CFC_11_ eq	3.24		2.63	0.01	3.00 × 10^−3^	1.32 × 10^−3^	0.25	6.24 × 10^−3^	6.49 × 10^−5^	0.34	2.19 × 10^−3^	3.98 × 10^−3^
OF	g NO_x_ eq	3.46		0.68	0.02	0.02	8.71 × 10^−3^	1.62	0.05	2.18 × 10^−4^	1.04	0.01	0.01
TA	g SO_2_ eq	8.70		4.13	0.05	0.04	0.03	2.05	0.13	4.69 × 10^−4^	2.23	0.02	0.01
FE	g P eq	0.85		0.08	5.61 × 10^−3^	2.02 × 10^−3^	1.39 × 10^−3^	0.18	3.86 × 10^−3^	1.02 × 10^−4^	0.59	8.37 × 10^−4^	9.89 × 10^−4^
ME	g N eq	2.22	1.55	0.59	6.17 × 10^−4^	1.30 × 10^−4^	1.95 × 10^−4^	0.01	0.03	7.58 × 10^−6^	4.17 × 10^−2^	5.54 × 10^−5^	9.78 × 10^−5^
TET	kg 1,4-DCB	2.24		0.85	0.02	3.32 × 10^−2^	0.01	0.91	0.05	9.80 × 10^−5^	0.35	0.01	1.18 × 10^−3^
FET	g 1,4-DCB	79.65	50.07	4.83	0.16	0.66	0.04	8.29	0.18	2.92 × 10^−3^	15.36	0.03	0.03
MET	g 1,4-DCB	39.13	1.20	3.72	0.22	0.91	0.07	11.35	0.27	4.08 × 10^−3^	21.32	0.04	0.04
MRS	g Cu eq	2.46		0.95	5.81 × 10^−3^	0.93	2.26 × 10^−3^	0.22	0.02	3.02 × 10^−4^	0.34	3.55 × 10^−4^	3.83 × 10^−4^
FRS	kg oil eq	0.66		0.07	2.67 × 10^−3^	2.75 × 10^−3^	1.17 × 10^−3^	0.40	0.02	3.04 × 10^−5^	0.16	3.00 × 10^−3^	4.70 × 10^−3^
HT, c	×10^−10^ CTUh	2.20		1.79	7.1 × 10^−3^	3.73 × 10^−3^	1.71 × 10^−3^	0.28	0.01	2.67 × 10^−5^	9.46 × 10^−2^	5.71 × 10^−3^	0.01
HT, nc	×10^−10^ CTUh	4.33		3.13	0.02	0.05	4.56 × 10^−3^	1.09	0.01	1.88 × 10^−5^	2.11 × 10^−2^	4.11 × 10^−4^	4.15 × 10^−4^

**Table 8:** Environmental characterization of starch bioadhesive oxidized with FeSO_4_ and H_2_O_2_.

Impact Category	Unit	Total	Emissions	Corn Starch	Water	FeSO_4_	H_2_O_2_	PVA	SDS	APS	VAc	BAc	Electricity	Heat, Steam	Heat, Cooling
GW	kg CO_2_ eq	1.10		0.05	1.45 × 10^−4^	7.08 × 10^−5^	6.98 × 10^−4^	0.04	3.70 × 10^−4^	1.00 × 10^−3^	4.94 × 10^−3^	0.30	0.56	0.11	0.01
SOD	mg CFC_11_ eq	1.27		0.38	8.32 × 10^−5^	3.88 × 10^−5^	2.48 × 10^−4^	0.02	1.46 × 10^−4^	3.28 × 10^−4^	0.54	0.11	0.15	0.06	1.59 × 10^−3^
OF	g NO_x_ eq	2.22		0.10	2.80 × 10^−4^	1.80 × 10^−4^	1.04 × 10^−3^	0.10	9.61 × 10^−4^	1.97 × 10^−3^	0.01	0.71	1.07	0.20	0.01
TA	g SO_2_ eq	4.06		0.59	6.02 × 10^−4^	2.59 × 10^−4^	1.68 × 10^−3^	0.13	3.61 × 10^−3^	4.48 × 10^−3^	0.02	0.90	1.95	0.42	0.01
FE	g P eq	0.34		0.01	1.31 × 10^−4^	5.74 × 10^−5^	2.18 × 10^−4^	0.01	1.53 × 10^−4^	2.22 × 10^−4^	1.11 × 10^−3^	0.08	0.13	0.11	6.08 × 10^−4^
ME	g N eq	0.11		0.09	9.73 × 10^−6^	4.08 × 10^−6^	4.68 × 10^−5^	7.5410^−4^	2.15 × 10^−5^	1.43 × 10^−5^	7.64 × 10^−5^	0.01	0.01	0.01	4.02 × 10^−5^
TET	kg 1,4-DCB	1.05		0.12	1.26 × 10^−4^	3.05 × 10^−4^	8.90 × 10^−4^	0.06	1.28 × 10^−3^	3.64 × 10^−3^	2.88 × 10^−3^	0.40	0.38	0.07	0.01
FET	g 1,4-DCB	12.98	0.61	0.70	3.75 × 10^−3^	1.55 × 10^−3^	0.03	0.51	4.78 × 10^−3^	0.07	0.04	3.63	4.38	2.91	0.02
MET	g 1,4-DCB	16.83	0.01	0.54	0.01	2.29 × 10^−3^	0.04	0.70	0.01	0.10	0.06	4.98	6.24	4.04	0.03
MRS	g Cu eq	0.66		0.14	3.88 × 10^−4^	3.38 × 10^−5^	8.95 × 10^−4^	0.01	2.50 × 10^−4^	0.10	8.79 × 10^−4^	0.10	0.24	0.06	2.58 × 10^−4^
FRS	kg oil eq	0.52		0.01	3.90 × 10^−5^	1.95 × 10^−5^	2.49 × 10^−4^	0.02	1.29 × 10^−4^	3.02 × 10^−4^	1.18 × 10^−3^	0.18	0.26	0.03	2.18 × 10^−3^
HT, c	10^−10^ CTUh	5.80		2.57	3.43 × 10^−4^	2.80 × 10^−4^	4.43 × 10^−3^	0.17	1.88 × 10^−3^	4.09 × 10^−3^	0.01	1.21	1.38	0.18	0.04
HT, nc	10^−10^ CTUh	13.07		4.51	2.41 × 10^−4^	2.13 × 10^−3^	3.31 × 10^−3^	0.67	0.01	0.05	1.35	4.79	1.64	0.04	2.99 × 10^−3^

**Table 9:** Environmental characterization of starch bioadhesive oxidized with NaClO and ECH.

Impact Category	Unit	Total	Emissions	Corn starch	Water	NaClO	ECH	PVA	LSS	Tween80	APS	VAc	EPR	Catalyst	Electricity	Heat, Steam	Cooling Energy
GW	kg CO_2_ eq	1.82		0.09	9.38 × 10^−5^	3.90 × 10^−3^	6.90 × 10^−3^	0.05	8.60 × 10^−3^	1.83 × 10^−2^	4.43 × 10^−3^	0.42	1.04	0.04	0.12	0.01	2.63 × 10^−3^
SOD	mg CFC_11_ eq	1.45		0.78	5.37 × 10^−5^	4.78 × 10^−3^	3.89 × 10^−3^	0.02	0.03	4.27 × 10^−3^	1.45 × 10^−3^	0.15	0.38	0.01	0.07	1.47 × 10^−3^	7.64 × 10^−4^
OF	g NO_x_ eq	3.87		0.20	1.80 × 10^−4^	0.01	0.01	0.11	0.03	0.03	0.01	1.00	2.15	0.08	0.21	0.01	1.96 × 10^−3^
TA	g SO_2_ eq	6.21		1.23	3.88 × 10^−4^	0.01	0.02	0.15	0.06	0.05	0.02	1.27	2.81	0.13	0.45	0.01	2.29 × 10^−3^
FE	g P eq	0.59		0.02	8.46 × 10^−5^	2.17 × 10^−3^	1.79 × 10^−3^	0.01	2.21 × 10^−3^	4.41 × 10^−3^	9.81 × 10^−4^	0.11	0.30	0.01	0.12	5.64 × 10^−4^	1.90 × 10^−4^
ME	g N eq	0.26		0.18	6.28 × 10^−6^	2.30 × 10^−4^	1.85 × 10^−4^	8.65 × 10^−4^	0.01	3.01 × 10^−4^	6.31 × 10^−5^	0.01	0.02	0.03	0.01	3.73 × 10^−5^	1.88 × 10^−5^
TET	kg 1,4-DCB	2.18		0.25	8.11 × 10^−5^	5.64 × 10^−3^	8.69 × 10^−3^	0.06	1.67 × 10^−2^	1.12 × 10^−2^	1.61 × 10^−2^	0.57	1.09	0.07	0.07	8.54 × 10^−3^	2.27 × 10^−4^
FET	g 1,4-DCB	1409	1386	1.43	2.42 × 10^−3^	0.06	0.06	0.59	0.61	0.14	0.32	5.13	11.11	0.33	3.09	0.02	0.01
MET	g 1,4-DCB	61.74	33.11	1.10	3.38 × 10^−3^	0.09	0.09	0.80	0.34	0.20	0.44	7.02	13.74	0.49	4.28	0.03	0.01
MRS	g Cu eq	1.34		0.28	2.50 × 10^−4^	2.26 × 10^−3^	2.35 × 10^−3^	0.02	0.03	4.61 × 10^−3^	0.45	0.14	0.31	0.04	0.07	2.39 × 10^−4^	7.36 × 10^−5^
FRS	kg oil eq	0.81		0.02	2.51 × 10^−5^	9.90 × 10^−4^	2.23 × 10^−3^	0.03	2.37 × 10^−3^	8.40 × 10^−3^	1.33 × 10^−3^	0.25	0.44	0.02	3.17 × 10^−2^	2.02 × 10^−3^	9.03 × 10^−4^
HT, c	×10^−10^ CTUh	3.06		0.53	2.21 × 10^−5^	3.07 × 10^−3^	2.97 × 10^−3^	1.95 × 10^−2^	2.47 × 10^−2^	2.35 × 10^−2^	1.81 × 10^−3^	0.17	2.24	2.21 × 10^−2^	1.89 × 10^−2^	3.85 × 10^−3^	2.07 × 10^−3^
HT, nc	×10^−10^ CTUh	5.35		0.93	1.55 × 10^−5^	7.99 × 10^−3^	7.27 × 10^−3^	7.73 × 10^−2^	2.41 × 10^−2^	4.65 × 10^−3^	2.33 × 10^−2^	0.68	3.58	2.07 × 10^−2^	4.20 × 10^−3^	2.77 × 10^−4^	7.96 × 10^−5^

## Data Availability

Not applicable.

## Sample Availability

Not available.
